# Insights into SARS-CoV-2 Surveillance among Prison Populations in Mato Grosso do Sul, Brazil, in 2022

**DOI:** 10.3390/v16071143

**Published:** 2024-07-16

**Authors:** Liliane Ferreira da Silva, Luiz Carlos Junior Alcantara, Vagner Fonseca, Diego Frias, Marina Castilhos Souza Umaki Zardin, Gislene Garcia de Castro Lichs, Ana Olivia Pascoto Esposito, Joilson Xavier, Hegger Fritsch, Mauricio Lima, Carla de Oliveira, Larissa Domingues Castilho de Arruda, Livia de Mello Almeida Maziero, Ellen Caroline Rodrigues Barretos, Paulo Eduardo Tsuha Oshiro, Evellyn Fernanda Gimenes Mendes Menezes, Lucélia de Freitas Cardoso, Everton Ferreira Lemos, José Lourenço, Carlos F. C. de Albuquerque, Rodrigo Fabiano do Carmo Said, Alexander Rosewell, Luiz Henrique Ferraz Demarchi, Julio Croda, Marta Giovanetti, Crhistinne Cavalheiro Maymone Gonçalves

**Affiliations:** 1 Secretaria de Estado de Saúde, Campo Grande 79031-350, Mato Grosso do Sul, Brazil; enf.lilianesilva@gmail.com (L.F.d.S.); larissacastilhodocs@gmail.com (L.D.C.d.A.); mellolivia12@hotmail.com (L.d.M.A.M.); ellencarol1234567@gmail.com (E.C.R.B.); pauloedu.oshiro@gmail.com (P.E.T.O.); evellynj15@hotmail.com (E.F.G.M.M.); lucelia.enf2015@gmail.com (L.d.F.C.); crhismay@gmail.com (C.C.M.G.); 2 School of Medicine, Universidade Federal de Mato Grosso do Sul, Campo Grande 79070-900, Mato Grosso do Sul, Brazil; 3 Instituto René Rachou, Fundação Oswaldo Cruz, Belo Horizonte 30190-002, Minas Gerais, Brazil; alcantaraluiz42@gmail.com (L.C.J.A.); joilsonxavier@live.com (J.X.); hegger.fristch@gmail.com (H.F.); maurili15@hotmail.com (M.L.); 4 Climate Amplified Diseases and Epidemics (CLIMADE), Bairro Floresta 31110-370, Minas Gerais, Brazil; 5 Departamento de Ciências Exatas e da Terra, Universidade do Estado da Bahia, Salvador 40301-110, Bahia, Brazil; diegofriass@gmail.com; 6 Centre for Epidemic Response and Innovation, School of Data Science and Computational Thinking, Stellenbosch University, Stellenbosch 7600, South Africa; 7 Laboratório Central de Saúde Pública de Mato Grosso do Sul/SES/MS, Campo Grande 79031-350, Mato Grosso do Sul, Brazil; ninaumaki@gmail.com (M.C.S.U.Z.); glichs@hotmail.com (G.G.d.C.L.); ana.esposito@saude.ms.gov.br (A.O.P.E.); lhdemarchi@uol.com.br (L.H.F.D.); 8 Laboratório de Arbovírus e Vírus Hemorrágicos, Instituto Oswaldo Cruz, Manguinhos 21040-900, Rio de Janeiro, Brazil; oliveirasc85@gmail.com; 9 School of Medicine, Universidade Estadual do Mato Grosso do Sul, Campo Grande 79070-900, Mato Grosso do Sul, Brazil; everton.lemos@uems.com; 10 Faculdade de Medicina, Universidade Católica Portuguesa, Biomedical Research Center, 1649-023 Lisbon, Portugal; 11 Climate Amplified Diseases and Epidemics (CLIMADE), 1749-016 Lisbon, Portugal; 12 Coordenação de Vigilância, Preparação e Resposta à Emergências e Desastres (PHE), Organização Pan-Americana da Saúde/Organização Mundial da Saúde (OPAS/OMS), Brasília 25045-002, Federal District, Brazil; carloiscampelo@gmail.com (C.F.C.d.A.); saidrod@paho.org (R.F.d.C.S.); rosewelale@paho.org (A.R.); 13 Faculdade de Medicina, Universidade Federal de Mato Grosso do Sul, Campo Grande 79070-900, Mato Grosso do Sul, Brazil; juliocroda@gmail.com; 14 Department of Epidemiology of Microbial Diseases, Yale University School of Public Health, New Haven, CT 06510, USA; 15 Fundação Oswaldo Cruz, Mato Grosso do Sul, Universidade Federal de Mato Grosso do Sul, Campo Grande 79070-900, Mato Grosso do Sul, Brazil; 16 Department of Sciences and Technologies for Sustainable Development and One Health, Universita Campus Bio-Medico di Roma, 00128 Selcetta, Italy; 17 Oswaldo Cruz Foundation, Manguinhos 21040-900, Rio de Janeiro, Brazil

**Keywords:** COVID-19, SARS-CoV-2, prison, epidemiological monitoring, genomic monitoring

## Abstract

This study examines the epidemiological and genomic characteristics, along with the transmission dynamics, of SARS-CoV-2 within prison units I and II in Campo Grande, Mato Grosso do Sul, Brazil. Conducted between May and October 2022, it reveals how the virus spreads in the confined settings of prisons, emphasizing the roles of overcrowded cells, frequent transfers, and limited healthcare access. The research involved 1927 participants (83.93% of the total prison population) and utilized nasopharyngeal swabs and RT-qPCR testing for detection. Contact tracing monitored exposure within cells. Out of 2108 samples, 66 positive cases were identified (3.13%), mostly asymptomatic (77.27%), with the majority aged 21–29 and varying vaccination statuses. Next-generation sequencing generated 28 whole genome sequences, identifying the Omicron variant (subtypes BA.2 and BA.5) with 99% average coverage. Additionally, the study seeks to determine the relationship between immunization levels and the incidence of SARS-CoV-2 cases within this enclosed population. The findings underscore the necessity of comprehensive control strategies in prisons, including rigorous screening, isolation protocols, vaccination, epidemiological monitoring, and genomic surveillance to mitigate disease transmission and protect both the incarcerated population and the broader community.

## 1. Introduction

The World Health Organization’s declaration of the COVID-19 pandemic on 11 March 2020, highlighted the global challenge posed by the highly transmissible SARS-CoV-2 virus [1]. The disease it causes, COVID-19, manifests a range of symptoms from none to severe respiratory distress, complicating efforts to control its spread, especially in densely populated environments like prisons [2,3]. The significant proportion of infections that are asymptomatic, estimated to be between 40–45% [4,5,6], underscores the importance of contact tracing as an essential tool for managing outbreaks, particularly in settings where healthcare access is limited [7,8]. To effectively contain the virus, it is crucial to treat asymptomatic and symptomatic infections alike, employing measures such as isolation and contact tracing to prevent further outbreaks [9,10,11]. Prisons, with their characteristic overcrowding, poor ventilation, and substandard sanitary conditions, present a heightened risk for the rapid transmission of infectious diseases, including COVID-19 [12,13,14]. Brazil, having the world’s third-largest prison population, faces increased risk [15]. In such conditions, a single case in a Brazilian prison is estimated to infect up to 10 individuals, leading to a scenario where, in a cell housing 150 people, approximately 67% would be infected after 14 days, reaching 100% in 21 days [16].

Mato Grosso do Sul, with the third-highest prisoner per capita rate in Brazil—793.8 prisoners per 100,000 residents and operating at 171.79% above its capacity [17]—provides a critical case study for examining COVID-19’s spread within prisons. The state’s prisons, with their various degrees of exposure to external communities through activities such as family visits and legal consultations, along with the mobility of individuals within the prison system, highlight how SARS-CoV-2 can spread beyond the confines of incarceration, potentially affecting the wider community [18]. 

This study aims to address the knowledge gap regarding the epidemiological and genomic profile of COVID-19 in prison populations. Genomic sequencing plays a vital role in understanding the dynamics of the virus, informing public health strategies, and developing interventions. In the post-public health emergency phase in Brazil, with vaccines widely available, it becomes increasingly important to monitor and analyze the spread of COVID-19 in such vulnerable groups. The study focuses on the prison population of Mato Grosso do Sul, offering insights into the transmission dynamics and potential control measures to mitigate the spread within and beyond prison facilities. 

## 2. Materials and Methods

### 2.1. Study Settings

This research was carried out at two correctional facilities in Campo Grande, Mato Grosso do Sul: The Prison Unit I Closed Regime Men’s Penitentiary (PEMRFG I) and the Prison Unit II Closed Regime Men’s Penitentiary (PEMRFG II). Selection of these institutions was based on their identical architectural configurations, each featuring 110 cells distributed across five pavilions, delineated as follows: Pavilion 1 housing 26 cells with an additional 6 cells designated for disciplinary purposes, Pavilion 2 comprising 26 cells, Pavilion 3 also containing 26 cells alongside 6 disciplinary cells, Pavilion 4 encompassing 10 cells, and the Inclusion Pavilion, which includes 10 cells designated for inmates engaged in labor within the prison setting as a means to mitigate their sentences. Notably, Pavilion 4, identified as the Health Pavilion, was not utilized for health-related functions during the period of this study. The timeframe for the study, from May to October 2022, coincided with the end of the Public Health Emergency of National Concern declared in response to the COVID-19 pandemic in Brazil. This period also overlapped with the extensive dissemination of COVID-19 vaccines throughout the country, providing a unique context for evaluating the impact of vaccination on virus transmission within these penitentiary environments. 

As containment measures, we included isolation and contact tracing strategies to monitor exposure within the cells. The recommendation was to isolate the infected individuals. Initially, they were isolated in cells exclusively designated for this purpose. However, due to the number of cases, it became necessary to conduct isolation within the cells themselves, with 11 individuals isolated in exclusive cells (16.67%) and the remaining 55 individuals isolated within their own cells (83.33%). During the isolation period, activities in common areas and in-person visits were also suspended for all cellmates. Following confirmation, isolation was carried out, and contact tracing was conducted—effective strategies to prevent the spread in the prison environment, interrupting further transmission and ending outbreaks. Prison staff and field team members were tested every seven days, but the results were not included to focus on the prisoner population. 

### 2.2. Ethical Considerations

Ethical approval for the study was obtained from the Research Ethics Committee of the Federal University of Minas Gerais on 20 January 2021 (Opinion No. 4,504,785, CAAE 32912820.6.1001.5149). Informed consent was mandatory for all study participants. Consent was documented using a standardized form, with alternative methods such as fingerprinting employed for those unable to sign, witnessed by a third party. Refusal to participate or non-consent resulted in exclusion from the study. The recruitment process involved clear communication of the study’s scope. Compliance with safety measures was ensured in collaboration with prison authorities. In line with health regulations, confirmed cases were reported to the relevant health officials, with strict data confidentiality maintained.

### 2.3. Data Collection and Management 

Data were collected through a multi-step process. Initially, participants provided informed consent and then completed clinical and sociodemographic questionnaires. These forms were digitized and promptly uploaded to a secure online server. Data storage was facilitated using the REDCap^®^ system (https://projectredcap.org/), which allowed for controlled access by team members through individual logins to ensure confidentiality. Participant anonymity was maintained by coding each dataset upon entry, effectively masking any identifying information. Additionally, vaccination statuses were verified against the National Immunization Program Information System (SI-PNI). The vaccination breakdown among the participants was as follows: approximately 51% received the CoronaVac vaccine (Sinovac Biotech Ltd., Beijing, China), about 25% were administered the AstraZeneca vaccine (AstraZeneca, Cambridge, UK), around 17% received the Pfizer vaccine, and approximately 7% were given the Janssen vaccine (Johnson & Johnson Innovative Medicine, Beerse, Belgium). This protocol was implemented to ensure the secure and confidential management of participant data throughout the study.

### 2.4. Sample Collection and Molecular Screening 

The data collection for this study was conducted in two stages at the Prison Unit I and II Closed Regime Men’s Penitentiaries (PEMRFG I and II), with daily testing of approximately 40 to 55 individuals. The initial data collection phase for PEMRFG I took place from 3–25 May 2022, and then from 22 August to 6 September 2022, with an 88-day interval between phases. For PEMRFG II, data collection occurred from 14 June to 1 August 2022, and then from 9 September to 3 October 2022, with a 39-day break in between. Nasopharyngeal swabs were taken from individuals based on their symptoms, which were classified as either asymptomatic or symptomatic, using clinical definitions [18]. The collected samples were transported under refrigeration to the Central Laboratory of Health of the State of Mato Grosso do Sul (LACEN-MS) for molecular screening, with results made available within 48 h. At LACEN-MS, nasopharyngeal swabs were submitted to viral RNA extraction using the QIAamp Viral RNA Kits (QIAGEN, Hilden, Germany) according to the manufacturer’s instructions and subsequently to multiplex real-time PCR using the Allplex 2019-nCoV assay (Seegene, Seoul, Republic of Korea) [19]. This assay, which targets the envelope (E), RNA-dependent RNA polymerase (RdRp), and nucleocapsid (N) genes, was provided by the Brazilian Ministry of Health (BrMoH). Upon confirmation, samples were collected from each contact exposed to the confirmed index case (cellmates of an index case with a positive molecular diagnosis for SARS-CoV-2) on the seventh day after the initial collection. Confirmed cases of SARS-CoV-2 were reported via the e-SUS Notifica platform and communicated to the prison health team. Samples were collected for both diagnostic purposes and whole genome sequencing analysis.

### 2.5. Whole Genome Sequencing and Phylogenetic Analysis

For genomic sequencing, the Oxford Nanopore technology was used as previously described [20]. The selection of samples for sequencing was based on specific criteria, including a Ct value (≤30) and the availability of comprehensive epidemiological metadata, such as the date of sample collection, sex, age, and municipality of residence. The preparation of SARS-CoV-2 genomic libraries involved the use of nanopore sequencing technology. The process began with complementary DNA (cDNA) synthesis, utilizing the SuperScript IV Reverse Transcriptase kit (Invitrogen, Carlsbad, CA, USA) according to the manufacturer’s instructions. Following this, the generated cDNA was subjected to multiplex PCR sequencing with the Q5 High-Fidelity Hot-Start DNA Polymerase (New England Biolabs, Ipswich, MA, USA) and a set of specific primers designed by the ARTIC Network for sequencing the complete SARS-CoV-2 genome, as detailed by the Artic Network version 3 [21]. The PCR conditions employed have been previously reported [20]. All experimental procedures were performed within a biosafety level-2 cabinet to ensure safety and prevent contamination. The resulting amplicons were purified using 1× AMPure XP beads (Beckman Coulter, Brea, CA, USA) and quantified using a Qubit 3.0 fluorimeter (ThermoFisher, Waltham, MA, USA) with the Qubit dsDNA HS assay kit (ThermoFisher, Waltham, MA, USA). The DNA library preparation then proceeded using the ligation sequencing kit LSK109 and the native barcoding kit (NBD104 and NBD114, Oxford Nanopore Technologies, Oxford, UK). These prepared sequencing libraries were loaded onto an R9.4 flow cell (Oxford Nanopore Technologies, Oxford, UK) for sequencing. To monitor and detect potential contamination, negative controls were included in each sequencing run, achieving a mean coverage of less than 2%. The sequencing data were processed using Genome Detective [22]. Lineage assignment was conducted using the Phylogenetic Assignment of Named Global Outbreak Lineages tool (PANGOLIN, Cambridge, UK) [23]. The sequences generated in this study were compared to a diverse pool of genome sequences (*n* = 3036) sampled worldwide up to 15 October 2022. Due to the extensive amount of data available, the Subsampler tool (available at https://github.com/andersonbrito/subsampler (accessed on 12 June 2022)) was utilized for subsampling genomic data based on epidemiological time series data [24]. ViralMSA [25] and IQ-TREE2 [26] were employed for aligning all sequences and conducting phylogenetic analysis using the maximum likelihood approach, respectively. The raw maximum likelihood (ML) tree topology was then transformed into a dated tree using TreeTime (https://treetime.readthedocs.io/) [27], which applied a constant mean rate of 8.0 × 10^−4^ nucleotide substitutions per site per year after the exclusion of outlier sequences. This comprehensive methodological approach facilitated the estimation of the number of viral transmission events between various regions of the world, contributing valuable insights into the spread and evolution of SARS-CoV-2.

### 2.6. Epidemiological Data Assessment

Given our current understanding of the COVID-19 pandemic, we expected that the incidence of infection would decrease with an increasing number of vaccine doses and increase with the age of the individuals. To explore these dependencies, we initially categorized prisoners who tested positive into five classes based on the number of vaccine doses received, ranging from zero (unvaccinated) to four doses. We then examined (1) whether the number of positive cases decreased consistently with an increasing number of doses, (2) the intensity of this decreases, serving as an indirect measure of cumulative vaccine efficacy, and (3) the probable causes for any deviations observed. To address potential biases due to the non-uniform distribution of prisoners across the five dose categories, we recalculated the incidence in each class as the Standardized Incidence Ratio (SIR), defined as the ratio of the observed to the expected number of cases rather than merely counting the number of positive cases per class. This method provides a more accurate comparison and understanding of vaccine efficacy across different groups. Subsequent analysis focused on (1) the regularity of the decrease in incidence rate with increasing doses, (2) the intensity of this decreases as an unbiased measure of vaccine efficacy, and (3) exploration of any irregularities. Furthermore, we introduced a new metric, the Positive Representativeness Index (PRI), to complement the standard SIR analysis. The PRI modifies the SIR by subtracting one, resulting in a value that is positive when the SIR exceeds one, zero when equal to one, and negative when less than one. A positive PRI indicates an over-representation of positive cases, suggesting higher susceptibility among that group compared to expected rates if the vaccine dose number had no effect. Conversely, a negative PRI indicates under-representation, suggesting lower susceptibility. A key aspect of our methodology is the ability to identify the minimum number of vaccine doses that significantly reduce a subject’s susceptibility in the transmission chain. This is achieved by determining the smallest number of doses that result in a negative PRI. To analyze the effect of age on the transmission chain within the prison, we conducted a detailed analysis of the age distribution among positive and negative subjects. After verifying that the distributions were unimodal, we calculated mean and median ages, along with their standard deviations, to assess if there was a significant shift towards older age groups among the positive cases. This analysis helps to determine if increased age correlates with higher susceptibility, as expected. Moreover, in examining the interdependence of two concurrent variables—age and number of doses—on susceptibility, we plotted the average number of doses against prisoner age and calculated the Pearson correlation coefficient. A linear regression analysis was also conducted to explore any potential relationships between these variables. 

## 3. Results

**Figure 1:**
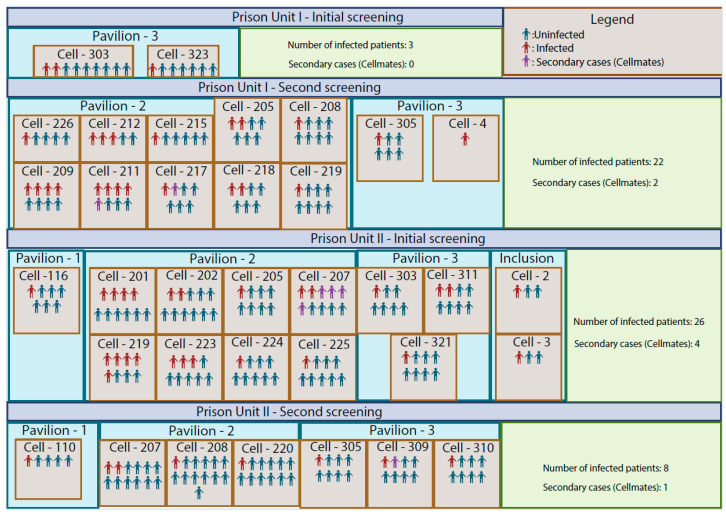
Epidemiological Distribution of SARS-CoV-2 in Prison Units I and II, Campo Grande, Brazil (May–October 2022). The figure maps the structural layout of the two prison facilities, delineating each cell block and pavilion to offer a detailed overview of the spatial configuration. For each unit, the figure shows the total number of screened sample environments during the study period.

The investigation was conducted at Prison Units I and II (Figure 1) in Campo Grande, Brazil, from May to October 2022. Specifically, the study enrolled a total of 1927 individuals who received a first assessment, and among these, an additional 181 contacts were evaluated, totaling 2108 samples. Out of these samples, 2040 were negative, and 66 were positive for SARS-CoV-2. Among the confirmed cases, 59 (89.39%) were identified as index cases, while seven (10.61%) were contacts of these index cases. This methodology allowed us to effectively trace contacts from identified index cases, enabling early detection of infections among inmates who were exposed to the virus, including asymptomatic cases. Such detection was crucial for controlling and breaking the chain of transmission. 

The testing strategy implemented in this study revealed a nuanced epidemiological profile of SARS-CoV-2 transmission within the prison population. Analysis of the data identified 59 primary (index) cases, which constituted 89.39% of the total confirmed infections. Additionally, through contact tracing efforts, an extra seven secondary cases were detected, representing 10.61% of the infections (Figure 1). This distinction between primary and secondary cases underscores the efficacy of contact tracing in identifying transmission chains within these settings. The study also delineated the temporal progression of the outbreak across the two prison units. Specifically, Prison Unit I witnessed a significant increase in cases, from 3 to 24, over an 88-day period between the initial and subsequent testing phases. Conversely, Prison Unit II experienced a reduction in cases, from 30 to 9, over a 39-day interval. Such temporal dynamics indicate the fluctuating nature of SARS-CoV-2 spread within these environments, impacted by various containment and mitigation strategies. A noteworthy observation from the second round of screenings was the considerable influx of new inmates, with a change of nearly 50% in the prison population—42.72% in Prison Unit I and 50.57% in Prison Unit II. This high turnover rate underscores the dynamic nature of prison populations and the potential for new introductions of the virus into the environment. Of the 220 prison cells, 33 recorded positive cases (Figure 1), illustrating the widespread nature of the virus within these facilities. The distribution of SARS-CoV-2 cases varied across different units, with Pavilion 2, the intake area, showing a higher frequency of new infections and an average of 8 people per prison cell (Figure 1). 

**Figure 2:**
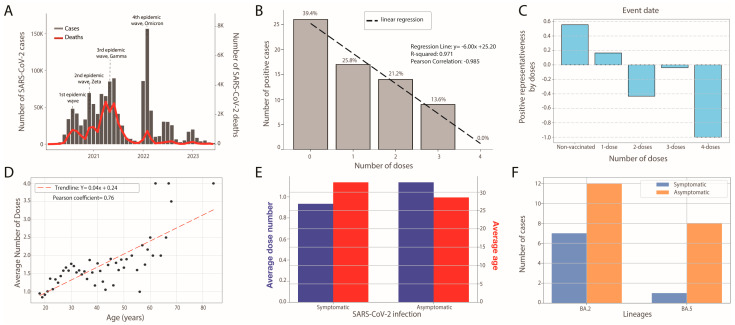
Epidemiological Dynamics of SARS-CoV-2 within Prison Units I and II in Mato Grosso do Sul. (**A**) Temporal distribution of daily SARS-CoV-2 cases and associated fatalities; (**B**) correlation between the number of positive cases and the number of vaccine doses administered among prisoners; (**C**) variation of the Positive Representativeness Index (PRI) with the number of doses; (**D**) correlation between number of doses and age of prisoners; (**E**) correlation between the average number of vaccine doses (in blue) received and the average age (in red), categorized by disease status categorized by host reaction to SARS-CoV-2 infection (symptomatic and asymptomatic); (**F**) correlation between the total number of SARS-CoV-2 cases per lineage (BA.2, BA.5), with cases colored based on clinical condition (symptomatic and asymptomatic infections).

The SARS-CoV-2 epidemic in Mato Grosso do Sul has seen four waves, with over 613,000 cases and 11,000 deaths by December 2023 (Figure 2A). The initial wave from March to August 2020 involved ancestral lineages (Figure 2A). From December 2020 to December 2021, the P.2 (Zeta) variant emerged, causing the second wave. The third wave followed due to the Gamma (P.1) variant, increasing cases and fatalities. However, with the Delta variant’s introduction in April 2021, numbers declined, with Delta becoming predominant by October 2021 (Figure 2A). The late November 2021 fourth wave, marked by the Omicron variant, peaked and fell quickly, hinting at the vaccination program’s effectiveness. Omicron’s high transmissibility significantly impacted the susceptible population (Figure 2A). In light of these evolving dynamics and the urgent need to adapt public health strategies, this study also aims to utilize whole genome sequencing to identify the SARS-CoV-2 variants circulating within the prison units. Additionally, it seeks to determine the relationship between immunization levels and the incidence of SARS-CoV-2 cases within this enclosed population (Figure 2).

In our investigation into the correlation between the degree of immunization and the incidence of SARS-CoV-2, we found a strong negative linear correlation (R^2^ > 0.97 and Pearson’s coefficient > 0.98) between the number of vaccine doses and the number of positive cases, as illustrated in Figure 2B. Specifically, our data indicate a 25% reduction in positive cases with each additional vaccine dose. Notably, individuals who had received four doses showed no positive cases, in stark contrast to the unvaccinated group, which represented approximately 40% (26 out of 66 cases) of all cases identified. This stark disparity underscores the vaccine’s protective efficacy and the potential benefits of expanding the vaccination protocol to include a fourth dose. It also highlights the urgent need to vaccinate unvaccinated prisoners. To address biases due to the non-uniform distribution of prisoners across five levels of immunization, as previously explained, we introduced the Positive Representativeness Index (PRI) in Figure 2C. This index provides an unbiased measure of susceptibility relative to the number of doses administered. Analyzing the PRI values (0.56, 0.17, −0.43−0.04, and −1) corresponding to increasing numbers of doses (0, 1, 2, 3, and 4), we observed a clear decreasing trend in susceptibility, with a notable exception at three doses which, while negative, deviates from the trend. Our approach confirms that the minimum number of doses required to effectively reduce susceptibility in the transmission chain is two, as indicated by the consistent decrease in PRI values from positive to negative with increasing doses. Further demographic analysis within the prison population revealed that while the youngest inmate is 18 and the oldest among positive cases is 47, the oldest among negative cases is 84. Notably, 95% of the population is under 44 years old, indicating a predominantly younger cohort. The mean age for positive cases is 29.5 years versus 31.1 years for negative cases—a slight but consistent shift towards younger individuals among positive cases, which is contrary to the expected trend of higher susceptibility in older individuals. Moreover, we explored the relationship between our primary independent variables, age and number of doses, plotting the average number of doses versus prisoner age and calculating the Pearson correlation coefficient (0.76). This strong correlation, displayed in Figure 2D, was anticipated given that the vaccination campaign initially targeted older individuals and was gradually extended to younger groups. This trend might also reflect the generally more hesitant attitude toward vaccination observed in younger individuals. In comparing symptomatic and asymptomatic cases, we found that asymptomatic individuals received, on average, 23% more doses than symptomatic ones (Figure 2E), suggesting the vaccine’s effectiveness in mitigating the severity of COVID-19. Additionally, symptomatic individuals were, on average, 4.12 years older than their asymptomatic counterparts. Within the studied population, the BA.2 variant was 2.1 times more prevalent and 3.3 times more severe than the BA.5 variant (Figure 2F). However, the age and dose differences between symptomatic and asymptomatic individuals did not reach statistical significance (*p*-value > 0.05), likely due to the small sample size of 66.

**Figure 3:**
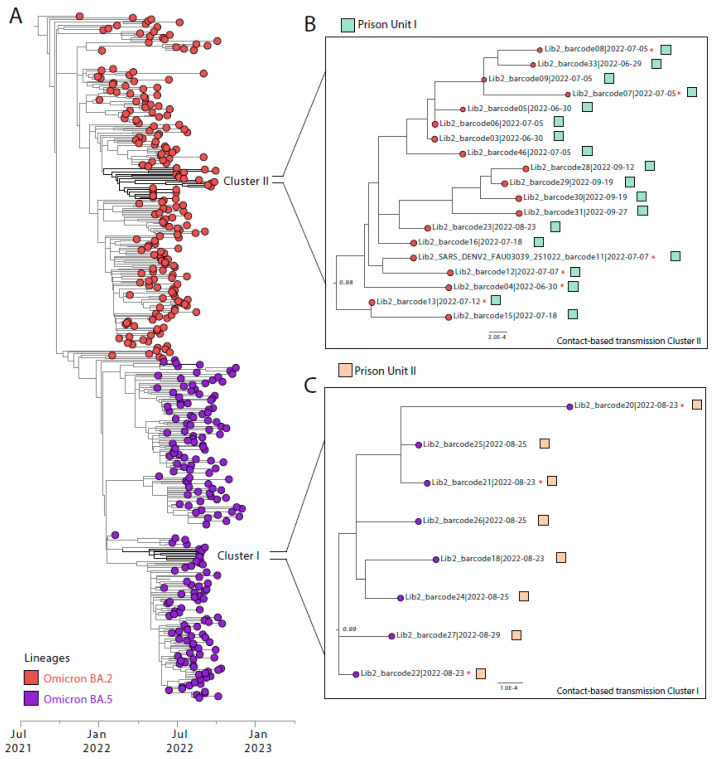
Phylogenetic Analysis of SARS-CoV-2 within Prison Units in Mato Grosso do Sul. (**A**) Maximum likelihood phylogenetic tree incorporating the 28 novel genome sequences generated in this study alongside 3036 reference sequences from the Omicron BA.2 and Omicron BA.5 SARS-CoV-2 variants. Colors indicate different lineages: Omicron BA.2 in red and Omicron BA.5 in purple (**B**,**C**). These panels depict the two distinct monophyletic clusters identified within Prison Units I and II, * respectively, highlighting clades suggestive of contact-based transmission.

The detected positive cases predominantly consisted of a younger demographic that did not require hospitalization and had minimal comorbidities. Among the confirmed cases, only 22.73% were symptomatic, exhibiting primarily cough, fever, headache, and sore throat. A subset of 42.42% (*n* = 28/66) of the detected positive cases, characterized by higher viral loads sufficient for genomic analysis, were genetically sequenced using nanopore technology. This process facilitated the generation of 28 complete genome sequences with an average coverage of 99%. Within this cohort, the Omicron variant emerged as the predominant strain, with the BA.2 lineage being the most frequently identified at 67.85%, followed by the BA.5 lineage at 32.15% (Figure 3 and Table 1).

Of the 28 genomes sequenced, 20 (71.43%) were from asymptomatic infections, and 8 (28.57%) were from symptomatic infections. Nine of these genomes were from prisoners who entered the unit less than 14 days ago, suggesting that their infections were acquired outside the prison. Our phylogenetic reconstruction revealed two transmission clusters, designated as Clade I and Clade II. Clade I include 19 genome sequences from prisoners infected with the Omicron BA.2 variant, while Clade II is composed of 8 genome sequences associated with the Omicron BA.5 variant.

## 4. Discussion

This study explores the complex epidemiological and genomic dynamics of SARS-CoV-2 transmission within the prison facilities of Mato Grosso do Sul, Brazil, offering essential insights for managing infectious diseases, including COVID-19, in confined settings. Our analysis identifies monophyletic clades and linear transmission chains, demonstrating how quickly the virus can spread through cell-to-cell transfers and frequent inmate movements, heightened by the inherent social dynamics of these environments [28,29,30,31,32]. Notably, we discovered an unusually high rate of asymptomatic infections—77.27%—likely due to comprehensive testing protocols that included asymptomatic individuals, highlighting their pivotal role in virus propagation. The timing of our study coincides with the prevalence of the Omicron variant and reveals a robust correlation between increased vaccination rates and a decrease in SARS-CoV-2 cases. Each additional vaccine dose administered resulted in a 25% reduction in cases. Remarkably, individuals who received four doses showed no positive cases, contrasting with the 26 noted among the unvaccinated [33,34,35,36,37,38]. This disparity underscores the vaccines’ efficacy and supports the potential benefits of integrating a fourth dose into the regimen to enhance immune resilience against the virus. Additionally, the importance of meticulous contact tracing, stringent testing protocols, and the implementation of comprehensive screening strategies tailored to the prison context cannot be overstated. These measures are crucial for disrupting transmission chains and managing outbreaks effectively [36,37,38]. Our study also emphasizes the necessity of ongoing genomic sequencing for continuous surveillance, which is vital for monitoring virus evolution and informing containment strategies. Together, the insights from this study underscore the need for a multifaceted approach to managing infectious diseases in prisons, including rigorous testing, extensive vaccination campaigns, and adaptive strategies tailored to each facility. These findings inform policy decisions and healthcare interventions critical for protecting the health of incarcerated individuals and the public, suggesting that policymakers consider implementing additional vaccine doses in the schedules for high-risk populations like inmates to maximize protection against COVID-19.

## 5. Conclusions

In summary, this study underscores the necessity of adopting a comprehensive and multifaceted strategy for the management of infectious diseases, including COVID-19, within prison environments. Key components of this approach include the implementation of robust testing protocols at the time of entry and exit, meticulous contact tracing, widespread vaccination efforts, and the enforcement of strict preventative measures. The detection of monophyletic clusters within the prison population not only sheds light on the dynamic transmission pathways of the virus but also underscores the vital importance of ongoing genomic sequencing. This level of surveillance is crucial for monitoring the virus’s evolution and dissemination, offering critical insights that are essential for the development of precise containment tactics. Such knowledge plays a pivotal role in informing policy decisions and health interventions aimed at safeguarding the health of incarcerated individuals and, by extension, the public at large from the extensive ramifications of COVID-19.

## Figures and Tables

**Table 1:** Epidemiological information from the sequencing of SARS-CoV-2 genomes in the PPL in the state of Mato Grosso do Sul.

Barcode ID (Date)	Prisonal Unit	Ct	Age	Reads	Coverage	Lineages	GISAID-ID
02|30 June 2022	PEMRFG II	23	20	8833	99.3	BA.5	EPI_ISL_18932422
03|30 June 2022	PEMRFG II	26	28	10,349	99.4	BA.2	EPI_ISL_18932444
04|30 June 2022	PEMRFG II	21	37	14,759	99.3	BA.2	EPI_ISL_18932421
05|30 June 2022	PEMRFG II	24	18	14,574	99.3	BA.2	EPI_ISL_18932443
06|5 July 2022	PEMRFG II	30	20	18,376	99.2	BA.2	EPI_ISL_18932424
07|5 July 2022	PEMRFG II	23	21	11,220	99.5	BA.2	EPI_ISL_18932446
08|5 July 2022	PEMRFG II	28	45	8605	99.3	BA.2	EPI_ISL_18932423
09|5 July 2022	PEMRFG II	28	28	10,122	99.2	BA.2	EPI_ISL_18932445
11|7 July 2022	PEMRFG II	26	28	1226	99.3	BA.2	EPI_ISL_18932426
12|7 July 2022	PEMRFG II	30	42	11,362	99.3	BA.2	EPI_ISL_18932425
13|12 July 2022	PEMRFG II	20	29	17,733	99.5	BA.2	EPI_ISL_18932447
15|18 July 2022	PEMRFG II	25	22	6266	99.3	BA.2	EPI_ISL_18932428
16|18 July 2022	PEMRFG II	28	19	4342	99.3	BA.2	EPI_ISL_18932427
18|23 August 2022	PEMRFG I	28	32	7203	99.3	BA.5	EPI_ISL_18932429
20|23 August 2022	PEMRFG I	29	26	10,434	99.4	BA.5	EPI_ISL_18932431
21|23 August 2022	PEMRFG I	23	27	13,823	99.4	BA.5	EPI_ISL_18932430
22|23 August 2022	PEMRFG I	23	26	17,071	99.2	BA.5	EPI_ISL_18932433
23|23 August 2022	PEMRFG I	25	21	3008	99.5	BA.2	EPI_ISL_18932432
24|25 August 2022	PEMRFG I	23	25	10,661	99.3	BA.5	EPI_ISL_18932435
25|25 August 2022	PEMRFG I	27	39	9969	99.2	BA.5	EPI_ISL_18932434
26|25 August 2022	PEMRFG I	29	45	11,444	99.2	BA.5	EPI_ISL_18932437
27|29 August 2022	PEMRFG I	22	34	13,700	99.6	BA.5	EPI_ISL_18932436
28|12 September 2022	PEMRFG II	23	46	12,771	99.3	BA.2	EPI_ISL_18932439
29|19 September 2022	PEMRFG II	28	27	8842	99.2	BA.2	EPI_ISL_18932438
30|19 September 2022	PEMRFG II	27	19	7928	99.3	BA.2	EPI_ISL_18932440
31|27 September 2022	PEMRFG II	19	18	14,835	99.3	BA.2	EPI_ISL_18932420
33|29 June 2022	PEMRFG II	30	21	10,976	99.2	BA.2	EPI_ISL_18932442
46|5 July 2022	PEMRFG II	33	26	1632	99.3	BA.2	EPI_ISL_18932441

## Data Availability

Newly generated SARS-CoV2- sequences have been deposited in GISAID under accession numbers EPI_ISL_18932420–EPI_ISL_18932447.
